# OMG! THEY LOOK JUST LIKE ME: EXPERIENTIAL LEARNING RELATED TO OLDER ADULTS AND HOMELESSNESS

**DOI:** 10.1093/geroni/igae098.2699

**Published:** 2024-12-31

**Authors:** Phyllis Greenberg, Isaac Asirifi Boateng, Sheila Moriarty, Claudia Burgos Zuniga

**Affiliations:** Saint Cloud State University, Saint Cloud, Minnesota, United States; Saint Cloud State University, Saint Cloud, Minnesota, United States; Saint Cloud State University, Saint Cloud, Minnesota, United States; Saint Cloud State University, Saint Cloud, Minnesota, United States

## Abstract

In 2023, nearly 1 in 4 people experiencing homelessness were over the age of 55. (National Alliance to End Homelessness 2023). As shocking as this number might be, it is estimated that the numbers of older adults who are homeless or at risk of homelessness are expected to nearly triple by 2030.To raise awareness in gerontology students a class studying research methods and a class studying policy teamed with social work students for Central Minnesota’s Project (Homeless) Connect. This a semester long project where students develop information for the larger community, city and state officials, as well as professionals about the growing numbers of older adults that are homeless or at risk of homelessness. In addition, to putting together and disseminating information students raise funds to (silent auctions, outreach to community donors, etc.) prepare basic necessity bags. The bags include everything from mini flashlight, rain poncho, shelf stable foods and a can opener among other things. Students also get socks, coats, and disposable underwear and other sanitary products that participants can choose from. The Project (Homeless) Connect event takes place annually and students work with participants to help them secure a variety of services from community vendors as well as learn more about them as individuals. Many of the participants have mentioned how they felt seen just by someone asking them their name and talking with them. This has been one of the more impactful experiences as well for students, faculty, staff and the community.

